# Efficacy and Safety of Microsomal Ferric Pyrophosphate Supplement for Iron Deficiency Anemia in Pregnancy

**DOI:** 10.7759/cureus.57108

**Published:** 2024-03-28

**Authors:** Ankita Srivastav, Shilpa Kshirsagar, Tejasvini Adhav, Gayatri Ganu, Alok Shah

**Affiliations:** 1 Periodontology, Shrimad Rajchandra Hospital and Research Centre, Dharampur, IND; 2 Gynecology, Lokmanya Medical Research Centre, Lokmanya Hospital, Chinchwad, IND; 3 Gynecology, Lifepoint Multispeciality Hospital, Pune, IND; 4 Pharmacology and Therapeutics, Mprex Healthcare Pvt. Ltd., Pune, IND; 5 Respiratory Medicine, Lung Injury Center, University of Chicago, Chicago, USA

**Keywords:** iron deficiency anemia, microsomal ferric pyrophosphate, ferritin, second trimester, hemoglobin, pregnancy

## Abstract

Introduction

Anemia during pregnancy is characterized by decreased hemoglobin levels. Iron deficiency poses a significant global health concern, especially in pregnant women, where increased iron demands are crucial for both maternal and fetal well-being.

Method

In the current study, we investigated the effectiveness and safety of 30 mg SunActive^TM^ Fe (Taiyo GmbH, Yokkaichi, Japan), emulsified microsomal^TM^ ferric pyrophosphate (EMFP) tablets in treating iron deficiency anemia in 27 second-trimester singleton pregnant women.

Results

Our study results demonstrated that hemoglobin levels increased significantly within 30 days of treatment and continued to remain higher than baseline throughout the study. Serum ferritin levels exhibited a 6.61-fold increase, maintaining elevated levels consistently. Serum iron also increased significantly by 46.9%. Additionally, symptoms such as nausea, breathlessness, dizziness, irritability, and heartburn were notably reduced, leading to improved quality of life. Subjects reported decreased overall fatigue, indicating an enhanced quality of life. Babies born during the study showed healthy birth weights, with uncomplicated deliveries. High treatment compliance of 99.5% underscored patient commitment to the study. Furthermore, the investigational product demonstrated a favorable safety profile, with only two mild adverse events observed, unrelated to the treatment.

Conclusion

These findings suggest that EMFP could be a valuable therapeutic option for managing iron deficiency anemia in pregnant women, promoting better maternal and fetal outcomes. Further research with an increased sample size is warranted to delve into the underlying mechanisms behind these positive outcomes, nonetheless, our study provides a promising foundation for addressing this critical health issue.

## Introduction

Anemia is a hematological condition marked by iron deficiency, reduced hemoglobin (Hb) levels, and low red blood cell (RBC) count [1,2]. It poses a serious global health threat, particularly prevalent in pregnant women due to increased iron demands and erythrocyte mass [3]. Anemia during pregnancy is categorized into mild (10-10.9 g/dL), moderate (7-9.9 g/dL), and severe below 7.0 g/dL [4]. National Family Health Survey 5 (NFHS) data (2019-2021) reveals a 52.2% prevalence of anemia during pregnancy, with rural areas showing higher rates [5,6]. Undetected and untreated iron deficiency anemia (IDA) adversely affects fetal development and maternal health, manifesting symptoms such as pallor, fatigue, and dizziness. Research links anemia to complications such as premature delivery, low birth weight (LBW), preeclampsia, postpartum maternal sepsis, hemorrhage, and maternal and child mortality [7,8].

IDA treatment includes oral and intravenous (IV) iron replacement. Oral iron, the mainstay of treatment, is convenient, inexpensive, and effective for stable patients. Various oral iron salts are in clinical use such as ferrous fumarate, gluconate, sulfate, etc. However, long-term treatment is limited by taste changes and gastrointestinal symptoms, necessitating frequent adjustments, prescription changes, non-adherence, or discontinuation. A systematic review revealed that gastrointestinal side effects pose the greatest challenges [9-11]. While IV iron is rapidly distributed, it does not lead to a faster increase in Hb levels and is linked to adverse effects, such as nausea, anaphylaxis, and extravasation [12].

Recently, researchers and clinicians have shown growing interest in developing alternative compounds with improved tolerability and higher bioavailability, which claim superior gastrointestinal tolerability, such as chelated ferrous forms or oral iron nanoparticles [9,12].

Therefore, it is crucial to identify the most suitable iron form and determine the optimal treatment duration to effectively replenish iron stores, restore normal Hb levels, and minimize side effects in a more assured and appropriate manner. The current trial aims to assess the safety and efficacy of 30 mg SunActive^TM ^Fe (emulsified microsomal^TM^ ferric pyrophosphate, EMFP) tablets in second-trimester singleton pregnant women with IDA, addressing crucial medical needs and providing a more appropriate and assured treatment strategy.

## Materials and methods

Study design

The current study, an open-label clinical investigation, aimed to assess the efficacy and safety of an iron supplement in second-trimester, singleton pregnant women with IDA. The patients were recruited after the ethics committee approval from the Institutional Ethics Committee of Lokmanya Medical Research and the Royal Pune Independent Ethics Committee. The study Clinical Trials Registry- India (CTRI) registration number is CTRI/2022/11/047376 (registered on: 17/11/2022). The study was conducted as per the approved protocol, declaration of Helsinki, and Good Clinical Practices guidelines. We screened 30 subjects from two study sites and got 27 complete patients for analysis. The data were collected from the period of December 2022 to June 2023 from the research sites.

Investigational product details

The investigational iron supplement tablet ingredients are depicted in Table 1.

**Table 1:** Ingredients of emulsified microsomal ferric pyrophosphate (EMFP) tablets

Sr. No.	Name of Ingredients	Composition
1.	Iron (ferric pyrophosphate)	30 mg
2.	Vitamin C	50 mg
3.	Vitamin B12	0.75 mcg
4.	Folic acid	250 mcg
5.	Glycine	10 mg

SunActive^TM^ Fe (Taiyo GmbH, Yokkaichi, Japan), an advanced microsomal^TM^ ferric pyrophosphate, is a highly micronized (0.3-0.5 microns) and bioavailable form of encapsulated iron. Its proprietary technology ensures that free iron does not come in contact with intestinal mucosa, guaranteeing efficacy and no side effects. Previous studies have shown that oral supplementation with SunActive^TM^ Fe was efficacious with no reported side effects and excellent patient compliance [13,14].

The optimal size of a nanoparticle to be taken up by the M cells and transcytosed from the basolateral side is below 1 μm. SunActive^TM^ Fe, the smallest available ferric pyrophosphate, has a scientifically proven mechanism of absorption through M cells, rendering safety and patient compliance [15].

Inclusion criteria

Pregnant females of age between 18 and 40 years (both inclusive) whose ultrasound at screening indicated a pregnancy between weeks 13 and 20 (both inclusive) and confirmed the presence of a live, singleton, intrauterine fetus and dating were included. We enrolled primi- or multigravida females without any comorbidity into the study. Pregnant females with Hb levels between 9 and 10.5 gm/dL (both inclusive) and serum ferritin levels between 10 and 15 mcg/L (both inclusive) were included in the study. Patients able to give written informed consent, and those able to follow up through visits were included in the study.

Exclusion criteria

Pregnant women of <13 weeks and >20 weeks of gestation with a complicated pregnancy history or ongoing treatment for it and complications such as bleeding piles, excessive emesis, active peptic ulcer, diabetes, hypertension, eclampsia, hypothyroidism, hyperthyroidism, and multiple pregnancies were excluded from the study. Patients who wished to continue any concomitant therapy for treating IDA during the study period were excluded from the study. Patients not willing to provide consent or follow-up were not included in the study. Any condition from the investigator’s viewpoint that affected patient participation was also excluded from the study.

Study methodology

After a written informed consent process on screening visits, the patient's demographic details were recorded. Patients underwent clinical examination, followed by hematological and biochemical assessment.

Upon checking possible allergies with the investigational product, on baseline visit (day one), patients received one tablet of 30 mg oral EMFP once daily before lunch for 90 days.

Drug adherence was assessed throughout the study, and if missed dosing for > 3 consecutive days or the total missed dose was > 6 during the 30-day period, the patient was considered as a dropout. Patients were advised to continue the diet and exercise regimen as suggested by the investigator and were assessed for any adverse events during the study period.

Clinical improvement, vitals, Hb, ferritin level, adverse event profile, symptoms score (four-point ordinal scale was used, with the following scoring: 0 - None: no symptoms were reported; 1 - Mild: symptoms were present but did not interfere with daily activities; 2 - Moderate: symptoms were moderate and interfered with daily activities; and 3 - Severe: symptoms were severe and significantly interfered with daily activities). The fatigue severity score by assessing the Functional Assessment of Chronic Illness Therapy - Fatigue (FACIT-Fatigue) scale, rescue medication assessment, concomitant diseases/medication assessment, adverse events, and patient compliance were assessed on every follow-up (i.e., weeks 4, 8, and 12 (serum iron on baseline and end of the study) by counting the remaining tablets compared to those dispensed. After the completion of 12 weeks, the patients were asked to stop the study medication. Fetal weight and gestational age (weeks) were recorded. T-test was used to compare the efficacy parameters with baseline and results at 30, 60, and 90 days.

Sample size

A total of 25 patients were intended to be evaluated in this study; however, a total of 27 patients completed the study. This sample size was based on a biostatistical power analysis looking at prior published results with SunActive^TM^ Fe. Laganà et al. previously conducted a study on second-trimester pregnant women in Italy, with a sample size of 25 women per arm [13]. This sample size was considered sufficient for the current pilot study.

Statistical analysis

Statistical analysis has been done by using Statistical Product and Service Solutions (SPSS, version 10.0; IBM SPSS Statistics for Windows, Chicago, IL). Upon checking the normality of data, all parametric datasets were analyzed using Student's t-test, while the non-parametric dataset used the Wilcoxon signed-rank test. P value was significant at p < 0.05.

## Results

Demographic data

In this study, the age of the patients ranged from 19.00 to 35.00 years with the mean age being 27.37 yrs.

Assessment of changes in Hb levels

**Figure 1:**
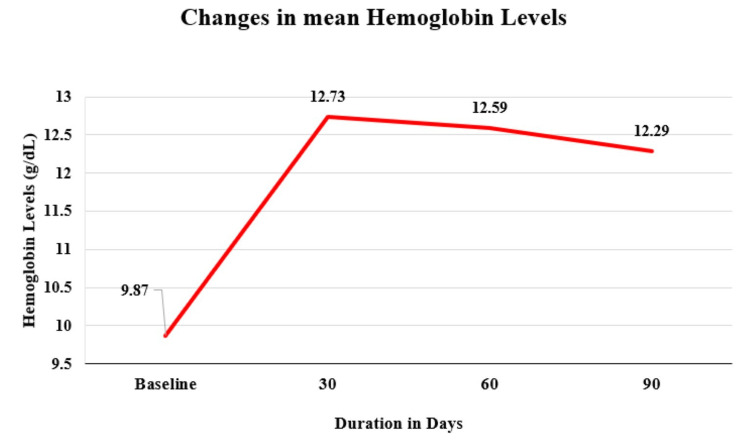
Changes in mean hemoglobin levels

At baseline, the mean Hb level was 9.87 gm/dL. Mean Hb levels showed a significant (p < 0.05) rise of 29% (▲ 2.86 g/dL), 27.6% (▲ 2.72 g/dL), and 24.4% (▲ 2.41 g/dL) after 30, 60, and 90 days, respectively, when compared to baseline (Table 2, Figure 1).

**Table 2:** Assessment of changes in mean hemoglobin levels Analysis done using Student's t-test. *Significant at p < 0.05.

Duration (days)	N	Mean hemoglobin (gm/dL) (± SD)
Baseline	27	9.87 ± 0.48
Day 30	27	12.73 ± 1.61
Day 60	27	12.59 ± 1.46
Day 90	27	12.29 ± 1.43
Mean diff (baseline–day 30) (p value)	27	*2.86 ± 1.71 (0.001)
Mean diff (baseline–day 60) (p value)	27	*2.72 ± 1.60 (0.001)
Mean diff (baseline–day 90) (p value)	27	*2.41 ± 1.53 (0.001)

Assessment of changes in serum ferritin levels

**Figure 2:**
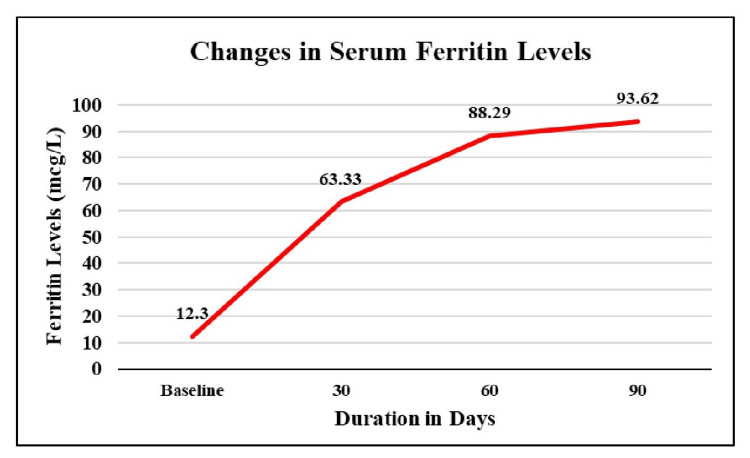
Changes in mean serum ferritin levels

At baseline, the mean serum ferritin level was 12.30 mcg/L. After 30 and 60 days, it showed a significant rise of 4.15 times (▲ 51.03 mcg/L) and 6.18 times (▲ 76 mcg/L), respectively. After 90 days, mean serum ferritin levels showed a significant rise of 6.61 times (▲ 81.32 mcg/L) from baseline (Table 3, Figure 2).

**Table 3:** Assessment of changes in mean serum ferritin levels Analysis done using Student's t-test. *Significant at p< 0.05.

Duration (Days)	N	Mean Serum Ferritin (mcg/L) (± SD)
Baseline	27	12.30 ± 01.96
30	27	63.33 ± 72.79
60	27	88.29 ± 92.63
90	27	93.62 ± 60.28
Mean diff (baseline–day 30) (p value)	27	*51.03 ± 72.32 (0.001)
Mean diff (Baseline–day 60) (p value)	27	*76.00 ± 92.99 (0.001)
Mean diff (Baseline–day 90) (p value)	27	*81.32 ± 61.03 (0.001)

Assessment of changes in serum iron levels

**Figure 3:**
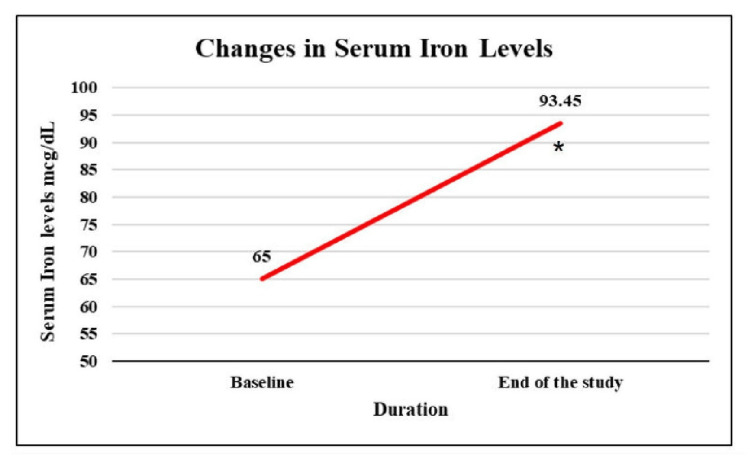
Changes in mean serum iron among study patients *p value < 0.05

At baseline, mean serum iron was 65.00 mcg/dL, and at the end of the study, it showed a significant rise of 46.9% from baseline (Figure 3).

Assessment of changes in fatigue

**Figure 4:**
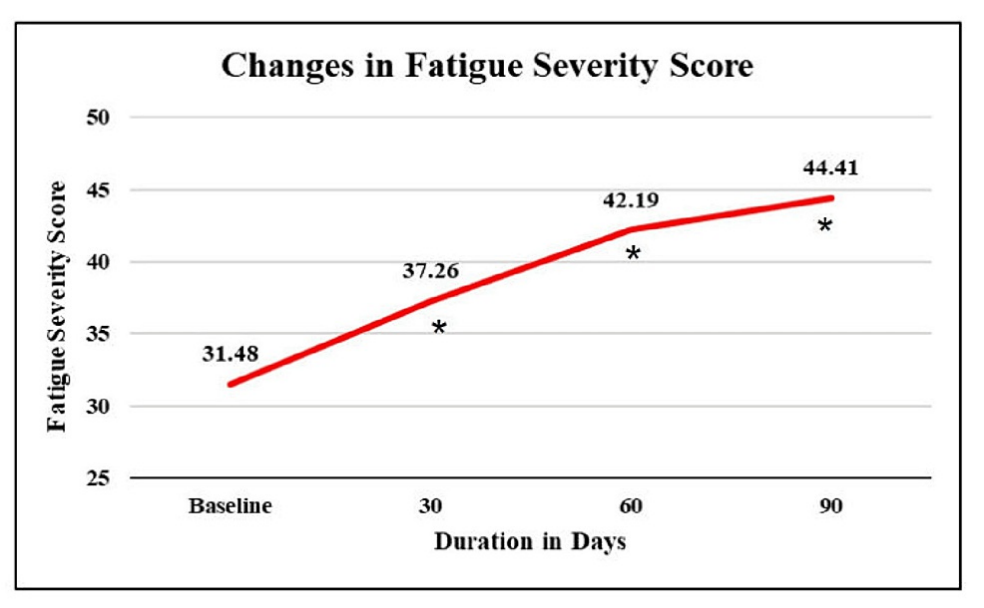
Changes in the mean fatigue severity score *p value < 0.05

At baseline, the mean fatigue severity score was 31.48 and showed a significant rise of 18.4%, 34%, and 41.1% after 30, 60, and 90 days, respectively (Figure 4). Hence, the overall fatigue score was increased gradually yet significantly over 90 days, indicating that subjects experienced less fatigue and improved quality of life.

Nausea

**Figure 5:**
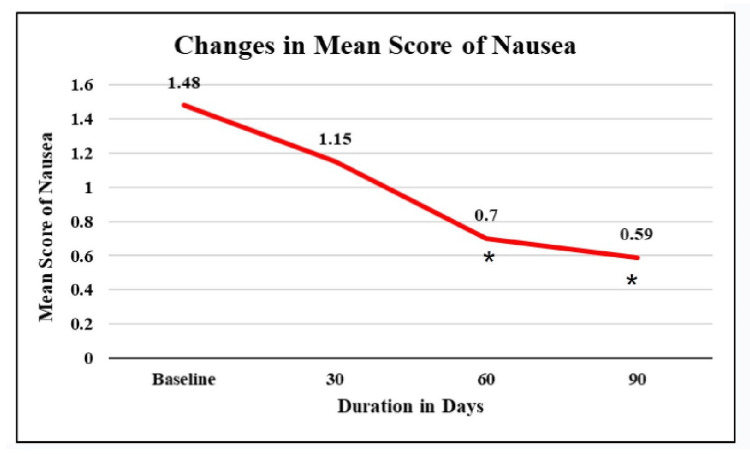
Changes in the mean score of nausea among study patients *p value < 0.05

According to this study, at baseline, the mean score of nausea was 1.48. After 30 days, there was an insignificant fall of 22.3% from baseline. It showed a significant fall of 52.7% and 60.1% from baseline after 60 and 90 days, respectively (Figure 5).

Assessment of changes in symptoms

In this study, we evaluated the severity of various symptoms every 30 days using a four-point ordinal scale of symptoms experienced by the patients, including breathlessness, dizziness, irritability, and heartburn.

At baseline, the mean score of breathlessness was 1.89. There was a significant fall of 56.6% (p = 0.001), 72.5% (p = 0.001), and 72.05% (p = 0.001) from baseline after 30, 60, and 90 days, respectively.

At baseline, the mean score of dizziness was 2.07. After 30, 60, and 90 days, a gradual yet significant fall was observed of 43.0% (p = 0.001), 62.8% (p = 0.001), and 78.7% (p = 0.001) from baseline, respectively.

At baseline, the mean score of irritability was 1.30, and it was reduced gradually over the course of the study. After 30 and 60 days, the mean score showed an insignificant fall of 14.6% (p = 0.441) and 28.5% (p = 0.075) from baseline, respectively. After 90 days, the mean score showed a significant fall of 48.5%(p = 0.030) from baseline.

At baseline, the mean score of heartburn was 0.70. After 30 and 60 days, it showed an insignificant fall of 5.7% (p = 0.833) and 15.7% (p = 0.535) from baseline. After 90 (p = 1.000) days, there was no significant change observed.

Assessment of changes in hematocrit levels

Mean hematocrit showed a significant rise of 18.8% from baseline (32.35 ± 3.76%) over the course of the study (37.55 ± 5.7)(p=0.002).

Assessment of birth weight

All patients proceeded to full-term pregnancies, characterized by uncomplicated deliveries. The average fetal birth weight recorded was 3.28 kg.

Assessment of treatment compliance

On day 30, compliance with treatment was 99.51%. The same trend was observed till the end of the study, which was 99.27% and 99.63% at days 60 and 90, respectively.

Assessment of profile of adverse events

Two adverse events, facial swelling and lachrymation, were reported, but they were not associated with the iron supplementation. All events were mild and resolved without any medications during the follow-up period.

## Discussion

IDA stands as the prevailing nutritional disorder globally and is frequently linked with unfavorable pregnancy outcomes. In the present study, subjects consumed a 30 mg EMFP tablet once daily for 90 days. Mean Hb levels were increased significantly by 24.4% at the end of the study compared to baseline. A significant increase of 2.8 g/dL was seen after 30 days of treatment with SunActive^TM^ Fe. At the end of the study, mean serum ferritin levels increased significantly by 6.61 times, and mean serum iron showed a significant rise of 46.9% from baseline. The nausea score was reduced by 60.1% among subjects at the end of the study, and the overall fatigue score increased gradually yet significantly by 41.1%, indicating that subjects experienced less fatigue, which can be correlated to improved quality of life. The symptoms of breathlessness, dizziness, irritability, and heartburn reduced gradually and significantly. The average birth weight was found to be 3.28 kg, indicating healthy children characterized by full-term and uncomplicated deliveries. Treatment compliance was almost 99.5% throughout the study. Only two adverse events not related to the iron supplementation were observed indicating the safety of the investigational product.

Research showed that the daily supplementation with 30 mg of micronized dispersible ferric pyrophosphate combined with 300 mg of alpha-lactalbumin was able to increase Hb, ferritin, serum iron, hematocrit, and RBCs in pregnant women more than 80 mg of ferrous gluconate, after 15 and 30 days of treatment [13]. In the current study, 30 mg of EMFP supplement also significantly increased Hb, ferritin, serum iron, hematocrit, and RBCs.

Treatment with SunActive^TM^ Fe significantly improved fatigue levels. The results were consistent with the previously conducted studies on pregnant women with normal iron status and third-trimester pregnant women with anemia [16,17].

In pregnancy, prioritizing early iron substitution ensures the well-being of mothers, improving their overall health and reducing potential risks.

Commonly used oral iron supplements, such as ferrous sulfate, ferrous gluconate, ferrous fumarate, and ferrous ascorbate, are known to cause adverse gastrointestinal effects. Research revealed that patients taking oral iron supplements reported gastrointestinal side effects in up to 60% of patients [18]. However, in the present study, there were no reported GI side effects. Moreover, we found that the investigational product was significantly effective in alleviating symptoms, including breathlessness, dizziness, irritability, nausea, and heartburn. An important result was seen anecdotally that every baby born from mothers enrolled in this trial was full term and of healthy birth weight. In a stark contrast birth cohort study involving 1,108 pregnant women who received iron supplementation, 57 preterm births were reported [19].

The limitations of the study are the small sample size, which might restrict the generalizability of the findings of the study. Addressing the limitation in future research is warranted to strengthen the evidence base.

However, 30 mg SunActive^TM^ Fe showed improvements in anemia status, indicating the effectiveness in treating IDA during the crucial trimesters of pregnancy.

Increased iron requirement is primarily due to the expansion of plasma volume and the growth of the fetus, which leads to physiological anemia in pregnant women. To ensure healthy pregnancy outcomes, it is crucial for pregnant women to maintain Hb levels above 11.0 g/dL, as recommended by the WHO [20]. Our findings highlight the increasing iron demand as pregnancy progresses and investigational products were sufficient to provide adequate iron.

This study shows that the encapsulated iron used is sufficient not only for efficacy in the second trimester in pregnant women but also is as good, if not better than conventional options, on the basis of literature reports. It completely nullified the biggest complaint of oral iron, that being of adverse effects that lead to lack of compliance.

The literature revealed that maternal Hb levels in the third trimester have an inverted U-shaped relationship with neonatal birth weight. Both low and high Hb levels were associated with low birth weight. Moreover, risks of LBW for gestational age followed an extended U-shaped curve, indicating that severe anemia and Hb levels above 13 gm/dL in the third trimester increased these risks [21]. In our study, the investigational product might have played a crucial role in maintaining optimal Hb levels (11-12 gm/dL), promoting healthy fetal development, and reducing the risk of adverse birth outcomes such as low birth weight.

## Conclusions

SunActive^TM^ Fe supplementation can serve as a choice of therapy for IDA in pregnancy, both therapeutically and prophylactically. The microsomal^TM^ technology of SunActive^TM^ Fe with the smallest particle size makes use of a unique mechanism exhibited by M cells. The results seen in this study suggest that SunActive^TM^ Fe is an attractive choice of treatment for IDA in pregnancy.
